# Viewing the image? Ultrasound examination during abortion preparations, ethical challenges

**DOI:** 10.1177/09697330211051009

**Published:** 2021-12-06

**Authors:** Marianne Kjelsvik, Ragnhild JT Sekse, Elin M Aasen, Eva Gjengedal

**Affiliations:** Department of Health Sciences in Aalesund, 8018 Norwegian University of Science and Technology (NTNU), Aalesund, Norway; Faculty of Health Studies, 155312 VID Specialized University, Bergen, Norway; Department of Obstetrics and Gynaecology, 60498 Haukeland University Hospital, Bergen, Norway; Department of Health Sciences in Aalesund, 8018 Norwegian University of Science and Technology (NTNU), Aalesund, Norway; Department of Global Public Health and Primary Care, 60518 University of Bergen, Bergen, Norway

**Keywords:** Early abortion, induced abortion, pregnancy, ultrasound, women’s reproductive health, decision-making, qualitative, moral sensitivity, four principles approach, ethics of care

## Abstract

During preparation for early abortion in Norway, an ultrasound examination is usually performed to determine gestation and viability. This article aims to provide a deeper understanding of women’s and health care personnel’s (HCP) experiences with ultrasound viewing during abortion preparation in the first trimester. Qualitative in-depth interviews with women who had been prepared for early abortion and focus group interviews with HCP from gynaecological units were carried out. A hermeneutic-phenomenological analysis, inspired by van Manen, was chosen. Thirteen women who were pregnant and considering abortion in their first trimester and 20 HCP, namely, 19 registered nurses and one medical doctor, were recruited from gynaecological units at six hospitals. The study was approved by the ethics committee (2014/1276). The essential meaning structure of ‘autonomy under pressure’ consisted of two themes that expressed the different experiences of both the women and the HCP, namely, expectations versus precautions and choice versus protection. The women and HCP expressed different attitudes before the consultation that affected their experiences of the ultrasound examination. While the women had expectations of a clarification based on their choice to either see or not see the ultrasound image, HCP seemed to be more concerned with predetermined rules that they believed would protect the women. Consequently, the basis for dialogue was not optimal, and women’s autonomy was under pressure. Health care personnel are ethically challenged during preabortion ultrasound examinations. Meeting the individual woman’s needs and respecting her autonomy during preparation for abortion requires sensitivity, involvement, and dialogue skills by health personnel. According to the woman’s desire to be informed about the possibility of viewing the image during the abortion preparations, a dialogue that is focused in this direction should arise before the examination.

## Introduction and background

Norwegian women do have the legal right to choose whether to terminate a pregnancy or not until the end of the 12th week of pregnancy.^1^ British^2^ and Norwegian^3^ clinical guidelines for the care of women requesting induced abortion recommend using ultrasound examination as a supplement when there is doubt about gestation or viability. At 7 weeks’ gestation, ultrasound images show an embryo that is approximately 9 mm, and the sound of a heartbeat can be perceived. At 10–11 weeks of pregnancy, the images might show a foetus with limbs that are moving.^4,5^

Prescriptions in clinical guidelines related to showing ultrasound images to women as part of abortion service provision differ. In the United Kingdom (UK), it is recommended that women are offered the opportunity to view their preabortion ultrasound scan.^2^ In Norway, viewing the image during abortion preparations is not covered in either the clinical guidelines^3^ or the general guidelines for ultrasound examinations in pregnancy.^6^ In the United States (US), several states regulate by law the provision of an ultrasound before an abortion. In some states, the providers are instructed to show and describe the image to the pregnant woman (mandatory viewing).^7^

Ethical challenges related to screening and the use of ultrasounds as part of maternity care were rarely debated in Norway until the 1990s. Since then, ethical questions and dilemmas related to foetal diagnostics have been on the agenda.^8^ In line with the democratization of health care, the focus in recent decades has moved from health professionals’ paternalism towards increased patient autonomy. Paternalism is in contrast with the democratic principle of the individual’s freedom and right to autonomy, which has become increasingly powerful (p. 230).^9^ Despite this, the idea of paternalism still has a great influence on health professions.

There is some research related to the experiences of women and health care personnel (HCP) during ultrasound examinations conducted at the time of abortion preparation in the first trimester. Due to the range of women’s experiences, it seems that many prefer to be given the choice of whether they want to view the image.^10–17^ Although UK women are given the opportunity to see the ultrasound image, the decision of whether they chose to see it and how doing so affects them varies. For some, looking at the foetus during abortion preparations is described as part of a final process and a form of closure rather than a way of attaching to the foetus.^14^ The ability to voluntarily see ultrasound images has been reported to help the decision-making process and satisfy women’s curiosity.^12,15,17^ A small proportion of US women with decisional uncertainty decide to continue the pregnancy after voluntarily viewing the ultrasound image. However, most US women report no impact on their abortion decision after viewing their ultrasound image.^16,18^

HCP’s perspectives of performing ultrasounds in preabortion settings in the UK and US are that an increased gestational age is associated with an increased emotional impact on women. HCP describe a desire to meet women’s needs and not cause emotional harm or influence the women’s decisions during examination. Thus, they present different views on whether women should be shown ultrasound images.^12,13,19^ Few, if any, studies from the Nordic context specifically relate to women’s or HCP’s experiences with ultrasound examination during abortion preparations in the first trimester.

Thus, the aim of this article is to provide a deeper understanding of this phenomenon, and the research questions were (1) How do pregnant women who are unsure about having an abortion, experience the ultrasound examination during the preparation for an abortion? and (2) How do HCP experience the ultrasound examination as part of the preparations for an abortion?

## Methodology and methods

An explorative design based on a hermeneutic-phenomenological approach^20^ was chosen due to the limited research available regarding preabortion consultations at gynaecological outpatient clinics. The data sources in this article derived from those used in two previous studies where women’s^21^ and HCP’s^22^ perspectives on the phenomenon of being ambivalent towards the decision to have an abortion were examined. Both from the women’s and the HCP’s perspective, we became aware of their feelings regarding either viewing or not viewing the ultrasound image during the preabortion consultation. The women and the HCP were asked about their experiences during the ultrasound examination as part of the preparations for an abortion. Thus, a deeper understanding of the phenomenon of viewing the image was sought during new analyses of the two data sets.

## Research context and participants

Between 10% and 18% of the women who arrive at a Western clinic to be prepared for terminating a pregnancy are ambivalent and undecided about what they will choose to do.^23–25^ In abortion care worldwide, the scope of practice for nurses and midwives has expanded.^26^ The first midwife-operated abortion care unit in Nordic countries opened in Sweden in 2009.^27^ In Norway, the preabortion ultrasound examination has traditionally been provided by a physician. In recent years, however, task sharing in regard to abortion care has developed, and specially trained nurses (RNs) in some gynaecological outpatient clinics have been delegated the entire preabortion consultation, including ultrasound examinations. In some of the units involved in this study, the nurses had been delegated such responsibility.

The inclusion criteria were women who were Norwegian-speaking, 18 years or older, in the first trimester of pregnancy and had not yet decided whether to have an abortion. Women who were too exhausted to be interviewed were not recruited. Thirteen women (18–36 years) who were pregnant were recruited from six gynaecological outpatient clinics at hospitals in urban and rural districts in South Norway. Recruitment took place at the end of the preabortion consultation. These women had been given more time for consideration than usual due to having expressed doubts during the preparations about the decision to end their pregnancy. The women’s reasons for considering an early abortion were complex and for various reasons related to psychosocial conditions. No foetal abnormalities were known or had been detected before or during the examinations.

For HCP, the inclusion criteria were nurses or medical doctors experienced in preabortion consultations. There were no exclusion criteria. Twenty HCP (24–60 years) were recruited from four of the same six hospitals from which the women were recruited. The HCP were all women. Most were well experienced with preabortion preparations, although a few were juniors. Nineteen were nurses, and one was a junior medical doctor. Their level of experience as HCP in a gynaecological unit ranged from 3 months to 33 years (mean 11.35 years) (Table 1).

**Table 1.** Characteristics and professional experiences of the participating health personnel.

Health personnel	*N* = 20
Gender	
Women	20
Men	0
Age	
24–35	6
36–50	5
51–60	9
>61	0
Experience in a gynaecological unit (years)	
<1	3
1–9	5
10–19	8
20–33	4

The following must be considered as strengths that support the transferability of the findings: both the women and HCPs were recruited from several hospitals and the sample of women had variations in age and the relationships with the men with whom they were pregnant. Some were first-time pregnancies, while others reported having had earlier pregnancies with birth and abortion experiences (Table 2).

**Table 2.** Characteristics and experiences of the participating women.

Women	*N* = 13
Age (years)	
18–20	4
21–29	5
30–36	4
Relationships with the man by whom they became pregnant	
Married or cohabitants	6
Established couple	3
Unclear or broken relationship	4
Pregnancy experiences	
First time pregnant	5
Had given birth previously	6
Had terminated a pregnancy previously	3
Had experienced spontaneous abortion	2

## Ethical considerations

The Regional Committee for Medical and Health Research Ethics (2014/1276) approved the study, which was designed in accordance with the Helsinki Declaration.^28^ Research permits were obtained from the leaders of all the study site organizations. During recruitment, the women who were asked to participate were chosen carefully, as some were recognized to be in a particularly vulnerable life situation. Both the women and the HCP were informed orally and in writing about the study, its purpose, that participation was voluntary and that they could withdraw from the study at any time before publication without explanation. All the participants provided their written consent.

## Data collection

In-depth interviews with women who were considering abortion and focus group interviews with HCP from gynaecological units were the data sources for this study. Data collection took place during 2015–2016. Most of the women were interviewed twice. The first interview with the women was carried out a few days after the preabortion consultation and before their final decision was made regarding whether to terminate their pregnancy. The duration of pregnancy when the first interviews were conducted with the women varied. Seven of the 13 women were in the sixth to ninth weeks of pregnancy, and the other six were between the 10th and 12th weeks. A follow-up interview was carried out some weeks after the 12th week of pregnancy was passed. Ten of the 13 women were interviewed twice, resulting in a total of 23 interviews. Approximately half of the women had terminated their pregnancy, and the others were still pregnant.

The individual interviews with the women lasted from 60 to 140 min (average 98 min) and were collected by the first author. Each of the three focus group interviews lasted approximately 100 min and were moderated by the first author and co-moderated by the last author. All the interviews were transcribed by the first author. All text material was organized into the NVivo 12^29^ software program after transcription to facilitate data management.

## Data analysis

Both sets of interviews were analysed by a hermeneutic-phenomenological approach inspired by van Manen.^20,30^ His recommendation to consider the text as a whole was followed by searching for essential aspects of the phenomenon in question, that is, the participants’ experiences related to the ultrasound examination during preabortion preparations. All the authors read through the interviews, and the first author suggested preliminary themes that were the subjects of discussion by the research team. Against this background, the themes were adjusted and synthesized until there was agreement on the final themes. Each interview with the women was analysed separately, and then preliminary themes for each of the two interviews were synthesized. The preliminary themes were compared and synthesized to form new preliminary themes across all 23 interviews. The focus group interviews with the HCP were also analysed separately, and preliminary themes from each interview were compared and synthesized to form a set of new themes. Finally, the preliminary themes from both data sets were compared to search for an essential meaning structure of the phenomenon, which in turn was divided into two final themes.

## Findings

The essential meaning structure was specified as ‘autonomy under pressure’. The meaning structure emphasized a difference in perceptions between the pregnant women and the HCP that contributed to the autonomy being perceived as pressured. Supported by their legal rights, the women believed that they could choose to see the ultrasound image or not, and they had different expectations about seeing it. HCP, however, were more concerned with taking precautions and thus protecting the women.

The final themes that were found to express the differences in the experiences of women and HCP were named expectations versus precautions and choice versus protection. These themes followed the timeline both before and during the ultrasound examination. The women’s experiences are presented prior to the HCP’s experiences in both findings.

## Expectations versus precautions

The women described several expectations related to either seeing or not seeing the foetus before the preabortion ultrasound examination. HCP, on the other hand, had a procedural focus and had been instructed by senior doctors to take precautions by turning the ultrasound screen away from their patients during preabortion preparations.

Before their preabortion preparation at the hospital, the women had various imaginings about and awareness of what they all described as a potential life. Some were actively seeking knowledge related to the pregnancy and development of the foetus, while others were trying to avoid dealing with it.

Some of the women took the stand that they wanted to see the ultrasound image if they were given the possibility. Reasons for wishing to view the image were based upon the intention to investigating what was the right decision for them. Some expected the whole situation to be more realistic if they were given the possibility to view the image. One said, ‘Maybe it will cause some emotion in me. Maybe I will feel like there is a baby there and that I can hear the heartbeat’. Another woman described her need to test her feelings as follows: ‘Actually, I wanted to see the image, to know if I felt anything’. Some participants based their desire to view the ultrasound images on their curiosity. One participant said, ‘Of course I wanted to see it because I was curious’.

Others decided that they did not want to view the image or hear a possible heartbeat during the ultrasound examination to protect themselves and not make it harder to choose abortion. One of the women who had given birth described, ‘When you get the ultrasound examination and you see it for the first time, the sight does something to you. To listen to the heartbeat for example. Yes, you remember the first time you heard the heart sound; it’s like it is in you forever. This time, I did not want to hear it’. Another woman described her decision to not view as follows: ‘If I had to see the ultrasound image and there was a baby in there, it would have not worked for me; I would have had to keep it’.

At the beginning of the preabortion consultation, the HCP from all the wards in this study reported that they took precautions related to hiding the ultrasound image from the women. They had been advised and had agreed upon a ‘rule’, passed down by senior doctors, which stated that women who are being prepared for an abortion should not be given the opportunity to see the ultrasound image. The focus should be to go through with the procedure; thus, the screen should always be turned away from the woman during the examination. Some had an understanding that a discretionary assessment was permitted, while others strictly adhered to the rule: ‘They are not allowed to see the picture. I have been so informed by my colleagues’.

## Choice versus protection

To not be given the choice to view the image meant for the women that they felt either protected or disrespected. The HCP described conflicting values when they considered whether they should adhere strictly to the ‘rule’ of hiding the image or take a more pragmatic stand when women express a desire to view (and/or be given information related to) the ultrasound image. The ‘face away the screen practice’ was based on HCP’s values related to protecting the women from emotional distress and not influencing their decision-making.

During the ultrasound examination, some women were offered the opportunity to see the image by HCP. Others had asked for it and been given permission. Some women were relieved not to have been confronted with the foetal sound or image.

What stands out in the women’s descriptions was that being given the choice for an independent (autonomous) decision regarding whether to view the image or not was interpreted as a respectful and caring act by HCP. The women who were allowed to view the image and had chosen to do so had various experiences. Some described not being affected emotionally. One of them said, ‘It did not bother me to see it. It did not truly give me that many strong feelings’. Others were upset by the sight. One related that she was overwhelmed and started to cry after viewing the image in her 11^th^ week of pregnancy: ‘I saw the child and then I felt “No, I do not want to do it” [to have an abortion]. However, I knew it was the most rational thing to do’.

For most women, the screen was turned away. Some of them reported that they had asked to see the image but were not allowed to do so. The women’s assessments concerning the hidden screen differed. Not being given the opportunity to view the image was accepted by some, while others were provoked and/or felt disrespected. Those who accepted the screen being turned away from them interpreted it as a protection measure and that the HCP knew what was best for them: ‘I think the doctor did it because I was already unsure, it could have affected me if I saw it’. For others, being denied viewing the image was described as a provoking act that excluded the women from insight regarding their own body. One of them said, ‘I do not understand why the doctor should decide if I should view the image or not. It is a picture of my child, and it must surely be up to me whether I want to see the picture or not. At least when I know it is there’. Another woman described the feeling of disrespect when not being allowed to view the image: ‘I thought it was a little pity. “You cannot see yet”, the doctor said. Therefore, I thought that I would probably see it afterwards. However, I did not. I asked if everything was normal and it was, the doctor said’. The participant was approximately 30 years old and had both given birth and had an abortion before the current pregnancy that she was considering terminating. A few women reported that they chose to view the image without permission, for example, by bending forward and looking at the inverted screen or by sneaking a view of the printout after examination. Others chose to visit a private ultrasound clinic after being refused a view of the ultrasound image at the hospital.

The HCP expressed that they assumed that the women who did not ask to see the image gave tacit consent to turn the screen away during the ultrasound examination. However, sometimes women did ask or argue to be allowed to view the ultrasound image. Most of the HCP described these situations as challenging. One said, ‘I find it uncomfortable when they ask to see; I get a very uncomfortable feeling’. Usually, the HCP denied the women the right to view the image. They gave several reasons for this refusal. Some told the women that the ultrasound image was for medical use: ‘We do not show the ultrasound image. We do turn away the screen. We say it is for our own routine, that we will use it in the medical record’. They also argued that hiding the screen was related to protecting the women from emotional distress and not influencing their decision-making: ‘You try your best not to make it harder for the patient. You want to protect the patient and to take care of her as much as possible. That is why we do not show the image to them’.

The HCP’s knowledge of the view of a developed foetus was part of their concern. Based on their previous experiences, they warned the women against the sight at the screen. One reflected as follows: ‘I think maybe they are curious because they do not quite know what they are seeing. In addition, at least for those who are further along, it can be shown there moving. I do not think they are prepared for that’. One from another unit said:

‘Basically, they are not allowed to look at the ultrasound. It is only if they almost demand it themselves, then we cannot deny them. However, we always recommend them not to see. The reason is that we should not be involved in influencing the decision. Because then they see that it is a living foetus there’.

Another HCP from the same group added: ‘Doing so adds slightly more relationship to it. When they see that it is alive, they think, “What am I doing?”’. These reflections were supplemented by a nurse from another group, who said, ‘It may be further along than you think. You can believe you are six weeks, and then find out you are 12 weeks. It can be shocking’.

In situations when women wanted to view the image, the HCP described assessing several factors, such as the women’s condition, the gestational age, the number of foetuses and the reasons stated by the woman for wanting to view the image. One described her considerations as follows: ‘I make an assessment due to age and type and whether they have had children before. In addition, then I think, “Yes, you can probably be strong enough to see this image”’.

The HCP reported that in retrospect, they have wondered whether women had received insufficient information for decision-making when they had been denied seeing the image. The HCP’s concern was also related to whether they, as professionals, knew what was best for each woman, better than the woman herself. One shared her experiences as follows: ‘Some [women] say, “This is my body, and I will see. I must be allowed to assess this myself”. I think it is difficult. I feel that they have the right to see, if they start arguing with me’.

Both the women and the HCP agreed on the importance of establishing a dialogue before the ultrasound examination. One of the women was concrete in her advice to HCP: ‘I would have liked the doctor to ask me, “Are you sure you want to know? Will you change your mind if you know how big it is or how small it is?”’.

Nevertheless, such a dialogue might create new dilemmas for HCP, as one of them described: ‘I do show women the ultrasound image sometimes. However, I always ask, “Why do you want to see?” And then it becomes slightly problematic, because then I must assess whether that reason is good enough’.

In some of the units, the HCP had discussed if they were in general legally permitted to hide the ultrasound image from the women. Some argued that the image and the eventual printout were part of the patient’s medical journal and thereby part of her belongings.

## Discussion

The women and HCP expressed different attitudes before the consultation that affected their experiences of the ultrasound examination. While the women had *expectations of a clarification* based on their choice to see or not see the ultrasound image, the HCP seemed to be more concerned with predetermined rules that they believed would *protect* the women. Consequently, the basis for dialogue was not optimal, and *women’s autonomy was under pressure*.

In the findings, the encounters between the women being prepared for abortion and the HCP revealed three different practices. Women’s autonomy was under the heaviest pressure in *Practice 1*, where the women wanted to view the image, but the screen was faced away, and the women’s requests were rejected. The HCP justified their decision by saying that they wanted to protect the women from the sight of the foetus to avoid influencing their decision and to make it less burdensome on the women.

However, what does it mean to protect a woman who is considering abortion? What is the HCPs’ motive for protecting women from the sight of an ultrasound image? What do the HCP see that women must be protected against? Some of the HCP in this study expressed such a concern, which is in line with UK health care professionals’ refusal to show the ultrasound image.^12,13^ These HCP have experienced that the sight of the image might make it extra stressful for the woman to have an abortion. Additionally, the British HCPs’ intention to hide the image is to protect the women, and they emphasize the ethical principle of not harming the patient.

According to Norwegian patient record regulations,^31,^ ^§ 11^ patients have a legal right to access their medical record, provided that doing so cannot negatively affect the patient’s health.^32^ Although HCP argued that viewing the ultrasound image may negatively affect the woman’s health, it is nevertheless reasonable to regard the image as part of the woman’s record. If this is the case, then the woman may, if she so wishes, have the right to see the image during the abortion preparations. According to some of the women, such insight was important in regard to making an autonomous decision. Thus, refusing the woman’s own desire to see the image on the screen is synonymous with withholding medical information and could thus be seen as an expression of paternalistic disrespect (p. 231).^9^

In the consultation, the HCPs’ focus is examining and preparing the women for the abortion. The preparations include both an ultrasound examination that is mainly for determining the gestation of the foetus and providing procedural information for the woman. However, a number of women being prepared for abortion at Western hospitals have not made a final decision to terminate their pregnancy.^23–25^ The women in this study had some ambivalence regarding whether to terminate their pregnancy. For some of those who asked for permission to see the screen, doing so was part of gaining insight and information to clarify their choice. Hiding the image may work against its intended purpose and thus be contrary to the effort not to influence the women’s decisions. To treat all women being prepared for abortion as if they are already fully clarified and informed about making a health-related choice may not be in accordance with legal regulations of patients’ understanding and consent (p. 118).^9^ In the worst case, the HCPs’ decision not to allow the image to be displayed may prevent a woman from making an informed choice in accordance with her own values. At the same time, there is no doubt that a woman may experience feeling vulnerable when considering whether to terminate or complete a pregnancy. This is important to take into consideration and may be a challenging assessment for HCP. What HCP reported fearing was that the woman, after having watched the image, would find it more difficult to terminate the pregnancy. In line with a US study,^18^ the voluntary viewing of ultrasound images may cause a small proportion of women with decisional uncertainty to decide to continue their pregnancy.

The most common practice related to viewing the image that the interviewed women had experienced and that the HCP conveyed was *Practice 2*, in which the woman had not been involved in the decision to hide the screen. This can be a solution that is in accordance with the woman’s wishes, but in reality, the HCP do not know if the woman wants to see the image as part of her decision-making process. What is this practice based on? Why do HCP not ask the woman whether or not she wants to see the image?

Having respect for others’ choices of action means both a negative duty, that is, not trying to control others when they act autonomously, and a positive duty, that is, according to Wifstad (p.64),^33^ informing and facilitating conditions so that others are better able to make autonomous decisions. Not asking the woman if she wants to see the image on the screen can be an expression of a passive waiting attitude in that the HCP do not involve the woman in the decision to turn the screen away. However, the consequence will then be that the HCP do not take into account that respect is not limited to the right attitude but also involves action.^33^

The practice of turning the screen away from women during abortion preparations contrasts with the practice in Norway and other Nordic countries related to screening for foetal diagnostics in the first trimester. Researchers have reported that during these consultations, the image is usually shown to women.^27,34–36^ The turned-away screen also contrasts with the ultrasound examinations given at Norwegian private clinics in the first trimester, where the display of the image is linked to whether the woman pays, not whether she plans to cancel or complete the pregnancy.^4^ Here, one may wonder what this difference in practice is due to. The burden placed on the woman at the sight of the foetus is probably the same whether the woman sees it in a public hospital or in a private clinic.

In *Practice 3*, some women wanted to view the image and were offered to see, or they were allowed to see when they asked for it. According to our findings, some HCP experienced that being strict in following the rules and keeping the screen turned away, despite the women asked to see the image, could be ethically wrong. Therefore, on some occasions, the HCP broke the rules and made demanding individual ethical judgements. The HCP thus respected the woman’s autonomy despite having concern about the consequences. However, for some of the women in this study, the worries of the HCP were confirmed. van Manen, 20 writes that: ‘Technologies always modify and transform the worlds that are revealed through them’ (p. 309).^20^ Seeing the image made some of the interviewed women doubt whether it was right to have an abortion, while others found peace of mind about their decision to have an abortion. This outcome is consistent with US women,^37^ who have reported that the sight of the foetus on the screen affects them emotionally and increases the difficulty of choosing abortion. For others, doing so helps them reconcile their decision to terminate a pregnancy.

To seriously take into consideration that a woman has imaginings about the foetus before the consultations and thus desires clarification, we may ask if it is possible for HCP during abortion preparations to contribute to both clarification and protection from harm. If such a viewing comes without information from the HCP regarding what the image shows, it might be harmful. For the ambivalent woman, viewing the image during the preparations, including receiving careful information, will most truly be better in retrospect than being given permission to gain insight into one’s medical record.

In addition, we can imagine a fourth practice, although it is not supported by our data, that is, the woman does not want to view the image but is forced to because of a visible screen in front of her. Such a practice can also be considered as paternalism and thus undermine the principle of autonomy.

Dialogue with HCP is of significant importance when patients have to make autonomous decisions.^9^ Requirements for the content of the information are extensive. For example, individually tailored information is required, and such information must be repeated.^31, § 3-2^ Undoubtedly, the women in this study wanted their agency to be respected in relation both to the ability to view the image and the final decision of whether to terminate their pregnancy. However, there are also challenges for HCP during these encounters. At the same time as having agency related to decision-making, both the women and the HCP described the women as being vulnerable and easily swayed. For example, in the dialogue, the HCP have to take into account that a woman may understand being offered to view the image as a form of pressure. To meet this challenge, some UK researchers recommend informing women prior to the ultrasound examination: ‘You will have an ultrasound scan today. The screen will be faced away; there will be no sound. If you want to see the scan, you can; just let me know’. (p. 133)^13^

## Conclusion

HCP are ethically challenged during preabortion ultrasound examinations. The basis for dialogue is not optimal, and women’s autonomy is under pressure or even not supported. A communication-related care comprising dialogue skills, sensitivity, and involvement, while meeting the individual woman’s needs, is crucial. In addition, it seems to be essential that HCP respect women’s autonomy and meet the conditions for informed consent. According to the woman’s desire to be informed about the possibility of viewing the image during abortion preparations, a dialogue that is focused in this direction should arise prior to the examination.
